# Relatives' Experiences of Circulatory Death Following Out‐of‐Hospital Cardiac Arrest and Their Hypothetical Perspectives on Organ Donation: A Qualitative Study

**DOI:** 10.1111/jan.70603

**Published:** 2026-04-05

**Authors:** Gisela Vogel, Karin Hildebrand, Mattias Ringh, Eva Joelsson‐Alm

**Affiliations:** ^1^ Department of Clinical Science and Education, Södersjukhuset Karolinska Institutet Stockholm Sweden; ^2^ Centre for Resuscitation Science, Department of Clinical Science and Education, Södersjukhuset Karolinska Institutet Stockholm Sweden

## Abstract

**Aims:**

To explore relatives' experiences of the circulatory death of a family member following out‐of‐hospital cardiac arrest and to explore their hypothetical perspectives on being asked to decide about organ donation in this situation. This study forms part of a broader initiative undertaken to assess the prerequisites for implementing uncontrolled donation after circulatory death in Sweden with a focus on ethical issues.

**Design:**

A qualitative design, using semi‐structured interviews with open‐ended questions.

**Methods:**

Data were collected in Sweden between March 2024 and September 2024 and analysed using reflexive thematic analysis with an inductive, descriptive approach. Included were 13 bereaved relatives, four men and nine women.

**Findings:**

The analysis generated three themes: ‘Fluctuating between hope and despair’, which describes relatives' experiences of their family member's sudden cardiac arrest; ‘Exposed to vulnerability’, concerning their experiences of death and their perspectives on reasoning about the deceased person's wishes regarding donation; and ‘Balancing a major life change’, which involves experiences of facilitators and barriers to dealing with the loss.

**Conclusions:**

Awareness of the deceased family member's wishes regarding organ donation facilitates relatives' ability to support the deceased's known or presumed wishes. Compassionate and communicatively competent healthcare professionals influence relatives' capacity to reason about the deceased's wishes and cope with their experience of loss. Ensuring sufficient time for goodbyes and providing follow‐up may facilitate emotional processing and should be considered when implementing uncontrolled donation after circulatory death.

**Implication for the Profession:**

Policies and clinical practice should ensure that relatives are adequately informed and supported in reasoning on their family members' wishes regarding organ donation. To achieve this, nurses and other healthcare professionals require training in compassionate and empathetic communication strategies. Such training is essential both for guiding relatives through reasoning about their family preferences and for providing emotional support during the grieving process.

**Impact:**

What is already known?

○ Organ donation after sudden death in out‐of‐hospital cardiac arrest is a complex process that raises ethical issues concerning both the timing and the content of conversation with the deceased's relatives, as well as the relatives' capacity to reason about the deceased's wishes in the context of sudden death.
○ Knowing the wishes of the deceased person, personal beliefs, and receiving clear, direct, and honest information from nurses and other healthcare professionals prepares relatives to reason about the deceased's known or presumed wishes regarding organ donation.

What were the main findings?

○ The quality of nurses' and healthcare professionals' communicative competence and their behaviours influence relatives' ability to reason and reflect on organ donation.
○ Factors that facilitate family members' experiences of donation following out‐of‐hospital cardiac arrest and death include awareness of the deceased's wishes regarding organ donation, being able to see with their own eyes that their family member is dead, and having sufficient time to say farewell.
○ Follow‐up from health care services could have an impact on relatives' experiences and their processing of loss.

Where and on whom will the research have an impact?

○ This research has an impact on organ donation services, pre‐hospital emergency care, and emergency department operations. It can assist nurses and physicians in planning ethical and family‐centred care in cases of out‐of‐hospital sudden death and uncontrolled donation after circulatory death.

**Reporting Method:**

COREQ‐checklist.

**Patient or Public Contributions:**

No patient or public involvement in design, conduct or reporting.

## 1 Introduction

Death due to sudden cardiac arrest is one of the leading causes of death in both Europe and the United States (Wong et al. 2019). Organ donation following death after sudden cardiac arrest is possible if a specific form of organ donation is utilised, uncontrolled donation after circulatory death, Maastricht category II (Thuong et al. 2016). This definition refers to donation from individuals who die after an out‐of‐hospital cardiac arrest with unsuccessful cardiopulmonary resuscitation. After resuscitation attempts have been completed, death has been declared, and consent has been obtained, post‐mortem medical interventions can be initiated, and organ donation can take place (Thuong et al. 2016). Sweden has not yet introduced uncontrolled donation after circulatory death. Before implementation, several issues need to be investigated, including the consent procedure, organ‐preserving techniques, considerations of best interests, identification of areas of consensus on clinical protocols, and early, sensitive engagement with families (Georgiou et al. 2025).

Sweden operates a soft presumed consent system, implying that an individual is presumed to be a donor after death unless they have explicitly registered or in other ways communicated their wishes not to be a donor (European Federation for Organ Donation 2025). Health care professionals seek to determine the deceased's presumed wishes, whether previously expressed and known or unknown, with relatives playing a key role in the process. However, although eight out of 10 inhabitants are positive towards donation, only 18% have registered their wishes in the Swedish donation register (National Board of Health and Welfare 2026). Therefore, relatives are often involved to support the fulfilment of the deceased's preferences (European Federation for Organ Donation 2025). In 2024, a total of 233 deceased individuals proceeded to organ donation in Sweden, including 59 donations after circulatory death and 174 donations after brain death (National Board of Health and Welfare 2026). Previous research has identified potential candidates for uncontrolled donation after circulatory death in Sweden. This study suggests that implementing uncontrolled donation after circulatory death protocols could increase the number of potential organ donors by approximately 15–73 individuals annually, depending on the selection criteria applied (Af Geijerstam et al. 2019).

## 2 Background

Until now, protocols for uncontrolled donation after circulatory death have only been implemented in a few European countries: Spain, France, Italy, and the Netherlands (Domínguez‐Gil et al. 2016; Lomero et al. 2020). Results from over 1000 kidney donations after uncontrolled donation after circulatory death in Spain and France have shown that uncontrolled donation after circulatory death is estimated to increase the number of possible donors by 25% (Del Río et al. 2019). However, previous studies indicate that, with regard to short‐term graft survival, uncontrolled donation after circulatory death is less effective than both donations after brain death and controlled donation after circulatory death (Lomero et al. 2020). This suggests that careful organ selection is essential. In countries that have not yet introduced the method, practical challenges, strict inclusion criteria (Pionnier et al. 2024), and obtaining consent from relatives under time pressure are crucial factors that complicate implementation (Georgiou et al. 2025; Pionnier et al. 2024). There are known challenges when approaching families to speak about organ donation, which may be challenging in an out‐of‐hospital cardiac arrest situation. Such a vulnerable situation requires specific knowledge and skills among nurses and other healthcare professionals (Georgiou et al. 2025).

Losing a family member is one of the greatest stressors in life, and grief is a normal emotional reaction following such a loss (Szuhany et al. 2021). However, witnessing the out‐of‐hospital cardiac arrest of a family member puts relatives in an extremely stressful situation (Douma et al. 2023; Ingles et al. 2016; Zhang et al. 2024) and could cause long‐term complicated grief and an increased risk of negative impacts on mental health (Carlsson, Bremer, et al. 2022; Kristensen et al. 2017; Steffen et al. 2020) as well as an increased risk of physical disease and premature mortality (Carlsson et al. 2023).

Previous research into relatives' experiences of giving consent to other types of organ donation methods, such as controlled donation after circulatory death or donations after brain death, describes relatives as potentially ambivalent about consent due to the ambiguity of brain death, uncomfortable with conversations about donation requirements, and in need of support after a donation (Ma et al. 2021). However, knowledge of the deceased person's wishes, personal beliefs, and clear, direct, and honest communication from nurses and other healthcare professionals are all important factors in preparing relatives for decisions about donation (Lo et al. 2022; Sque et al. 2018).

Introducing novel donation processes in Sweden, such as uncontrolled donation after circulatory death, has the potential to increase the donor pool and thus help reduce the gap between the demand and supply of donor organs (Lewis et al. 2021). Insufficient availability of organs results in long organ‐transplant waiting lists and subsequent suffering and death among people in need of organs (Domínguez‐Gil et al. 2016). Knowledge about relatives' complex needs and challenges after being exposed to a loved one's sudden death after out‐of‐hospital cardiac arrest is a prerequisite for being able to introduce uncontrolled donation after circulatory death. Therefore, this study aimed to explore relatives' experiences of the circulatory death of a family member following out‐of‐hospital cardiac arrest, and to explore their hypothetical perspectives on being asked to decide about organ donation in this situation.

## 3 Methods

### 3.1 Design

A qualitative design using semi‐structured interviews was employed to explore participants' narratives. The aim of the study was addressed within a paradigmatic framework of ontological relativism and epistemological constructivism, which means that people construct meaning from their experiences and express this through their individual perspectives. To gain a deeper understanding of participants' experiences, Reflexive Thematic Analysis, as developed by Braun and Clarke (Braun and Clarke 2014; Braun and Clarke 2006), was used as the analytical tool for an inductive, descriptive analysis focusing on lived experiences. The Consolidated Criteria for Reporting Qualitative Research (COREQ): a 32‐item checklist for interviews and focus groups (Tong et al. 2007) was used to report the data (File S1).

### 3.2 Study Setting and Recruitment

Volunteer participants were consecutively recruited through advertising in local newspapers across Sweden and on the websites of various organisations, such as the Swedish Resuscitation Council and Karolinska Institute. Advertisements were also placed in the magazine *Status* published by the Swedish Heart and Lung Association, on Karolinska Institute's Facebook page and on bulletin boards in an emergency hospital in Sweden. Anyone interested in participating in the study contacted the researcher via email to obtain more information. If the person then chose to participate, an appointment for the interview was booked. The advertising took place between March and September 2024 and resulted in the recruitment of 10 participants. Additional recruitment was carried out during May and June 2024 through data extraction of people who had died from out‐of‐hospital cardiac arrest in an emergency room at a hospital in Sweden between 1 January 2022 and 30 June 2023. Contact information (name, address, and telephone number) of relatives was obtained from the deceased's medical record by a research nurse. Six eligible people were contacted by letter with information about the study and the possibility to opt out of further contact. One week later, one of the researchers made contact by telephone with the individuals who had not declined to be contacted and asked whether they would participate in the study. Four people answered the phone, and one declined the invitation. This resulted in three additional participants, bringing the total number of participants recruited between 25 March and 25 September, 2024, to 13.

### 3.3 Inclusion and Exclusion Criteria

Inclusion criteria were adults (≥ 18 years) who were bereaved relatives of a person aged 18–75 years who had died from out‐of‐hospital cardiac arrest and transported to an emergency department during ongoing cardiopulmonary resuscitation. Relatives of individuals who died due to suicide or trauma were excluded, since such cases would constitute exclusion criteria in an uncontrolled donation after circulatory death process in Sweden. The definition of relatives used in this study included partners/spouses, siblings, children, and close friends.

### 3.4 Data Collection

The interviews were conducted between March and September 2024. One open‐ended question concerning relatives' experiences of their family member's out‐of‐hospital cardiac arrest and death was asked. In addition, more specific questions were asked about their experiences of healthcare professionals' behaviour and treatment, awareness of the moment of death, and perspectives on being asked to decide about organ donation in this situation. The interview guide translated into English is provided in File S2. During and immediately after the interviews, the interviewer wrote short field notes about the context, nonverbal behaviours, and critical reflections on her own performance. All interviews were conducted face‐to‐face by the first author (female) at a location chosen by the participants: in the participants' home (11 interviews) or in a room at the hospital (2 interviews), 3–48 months after a family member's out‐of‐hospital cardiac arrest and death. During all interviews, only the participant and the interviewer were present. The interviews lasted for about 30–60 min, and all participants were interviewed once.

Before the interview, participants were informed that the interviewer was an intensive care nurse and researcher. They were also informed about the purpose of the study and were asked if they had any questions before starting. All participants signed a written consent form and received written information about the study with contact details for the research team. During the interviews, the interviewer strived to create an open climate of trust. The participants were informed that it was fine to cry or pause the interview if emotions became overwhelming. Almost all participants cried during their interview, but no one paused it. Data collection and coding continued until saturation was reached, meaning that no new data, additional insights, or patterns were identified during the analysis. None of the researchers were employed in any emergency department where the participants' family member had been pronounced dead, nor had they been involved in the care of the deceased person or their relatives. The interviews were audio‐recorded and transcribed verbatim by the first author. Transcription by the first author was conducted as a part of the analysis to become familiar with the data. All authors had access to the audio files and transcripts.

### 3.5 Data Analysis

The analysis was carried out according to reflexive thematic analysis (Braun and Clarke 2006 and Braun and Clarke 2014). The transcripts were read in their entirety by three of the researchers (GV, KH, and EJA) to make sense of the data. Thereafter, data were coded into initial codes and preliminary themes, which were then discussed within the group to identify patterns that captured something significant or interesting. The preliminary themes were reviewed, discussed, and modified several times within the research group, together with discussions about how themes and sub‐themes interacted and related to each other. Finally, three themes and six sub‐themes were formulated and defined.

No data analysis software tool was used when analyzing the data.

### 3.6 Ethical Considerations

Ethical approval was obtained from the Swedish Ethical Review Authority (2023–08161‐01 [February 26th, 2024] and 2024–02843‐02 [May 26th, 2024]). The written material given to participants included information about the study and associated risks. All participants were informed about the voluntary nature of their participation and their right to withdraw. Furthermore, all participants signed a written consent form and received a copy with contact information for the researcher. Strict confidentiality was maintained regarding the participants. No data could be traced back to any individual, and all data were stored in a locked cabinet at the research centre and on a secure database only available to the researchers.

### 3.7 Rigour and Reflexivity

To establish the trustworthiness of the study, the criteria of credibility, dependability, confirmability, transferability (Korstjens and Moser 2018; Lincoln and Guba 1985), and reflexivity were applied (Korstjens and Moser 2018). Credibility was strengthened through the participants' rich and detailed narratives of their experiences. Participants were selected to ensure variation in personal characteristics. Furthermore, investigator triangulation was employed; none of the researchers works in the emergency ward, and the researchers represented different professional backgrounds and experiences of organ donation as nurses and physicians in intensive care units. Additionally, two of the researchers are actively working on donation‐related issues. The diverse professional backgrounds and pre‐understandings of the researchers facilitated critical reflections on the findings from multiple perspectives during the analysis. To achieve dependability and confirmability, the research process adhered to the six phases by Braun and Clarke (2014). Each phase was systematically documented and continuously discussed within the research group to ensure consistency and transparency. The method section provides a detailed description of the participants and research context, allowing readers to assess the transferability of the findings to their own settings. Regarding reflexivity, the researchers' preconceptions, grounded in their different professional backgrounds, were openly discussed within the research team to minimize potential bias in data collection, analysis, and presentation. This reflexive dialogue contributed to a comprehensive understanding of the complex factors influencing relatives who lost a family member in out‐of‐hospital cardiac arrest and received the death notification in the emergency department.

## 4 Findings

A total of 13 formal interviews were conducted, with four men and nine women. For characteristics of participants and their deceased family members, see Table 1. The analysis of participants' descriptions of their experiences of their family member's out‐of‐hospital cardiac arrest and death, along with questions regarding reflections upon their stances relating to organ donation in this situation, generated three themes: ‘Fluctuating between hope and despair’, ‘Exposed to vulnerability’ and ‘Balancing a major life change’, along with six sub‐themes, descriptions of themes with illustrative quotes (Table S1). The themes are presented under three subheadings and quotations in the text reflect the voices of the participants. The quotations are translated into English and written in italics.

**TABLE 1:** Demographic characteristics of the participants and their deceased family member.

Participant	Sex	Age (yrs)	Relation to the deceased	Present in the emergency department	Deceased persons sex	Deceased persons age (yrs)	Cause of death	Time between death and interview (months)
1	Female	58	Wife	Yes	Male	58	Myocardial infarction	12
2	Female	77	Wife	Yes	Male	72	Cardiomyopathy	19
3	Female	54	Mother	Yes	Female	29	Pulmonary embolism	6
4	Female	71	Wife	Yes	Male	73	Unknown	27
5	Female	71	Wife	No (Covid)	Male	72	Unknown	30
6	Female	75	Wife	Yes	Male	75	Myocardial infarction	23
7	Male	40	Son	Yes	Female	70	Unknown	30
8	Female	68	Sibling	No	Male	56	Unknown	20
9	Male	54	Father	Yes	Female	29	Pulmonary embolism	8
10	Male	31	Son	Yes	Male	64	Unknown	13
11	Female	37	Wife	Yes	Male	37	Unknown	48
12	Male	39	Son	Yes	Female	62	Unknown	17
13	Female	58	Wife	Yes	Male	62	Unknown	3

### 4.1 Theme 1: Fluctuating Between Hope and Despair

Fluctuating between hope and despair was a description of participants' experiences of their family member's sudden cardiac arrest, giving insights into the seriousness of the situation. This was perceived as an extremely stressful and lonely situation that was influenced by the actions of others.

*He had run and sat on the stairs, I was glad he was home. It became so quiet so I looked at him and he was lying down by the stairs, I thought he was joking but then I shook him and he was completely gone*. (2)

Sensitive and empathic behaviours from the rescue personnel or other people who witnessed the incident were perceived as support.

*The rescue manager stayed for a very long time and talked to me, it felt very good*. (5)

Those who witnessed the cardiac arrest or found their family member after the cardiac arrest often perceived the seriousness of the situation, and some understood that the person was already dead. Experiences of death were based on observations such as empty eyes, grey skin, gurgling sounds from the lungs, or a long time without cardiopulmonary resuscitation. Some of the participants, albeit only a few, believed that cardiopulmonary resuscitation may have been stopped too soon or that the rescuers might not have performed it correctly.

Hope was often described as linked to the time until cardiopulmonary resuscitation, the length of time cardiopulmonary resuscitation was administered, and experiences where everything possible was being done to save their loved one's life. Hence, awareness of the negative consequences of prolonged cardiopulmonary resuscitation and insight into the risk of brain injury reduced hope and caused despair about the future.

*When I was standing there during the* cardiopulmonary resuscitation *I thought ‘I hope he wakes up’ or ‘I hope not’ because he was probably very brain damaged. You're like that all the time*. (1)

For some, the sense of hope remained until death was clearly stated. Some participants had been contacted by telephone from the emergency department and informed of the death of their family member, and they already knew the outcome before they arrived. However, this message may be experienced as difficult to understand and/or handle. In addition, some participants then became responsible for calling other relatives and therefore deciding how to inform them, which could be perceived as stressful. A sense of loneliness was described, both feeling alone in the situation when one was forced to call for help and start cardiopulmonary resuscitation until help arrived, and/or a more existential feeling of emotional loneliness, even when emotional and/or practical support was received.

### 4.2 Theme 2: Exposed to Vulnerability

Participants described that the notification of the death delivered by healthcare professionals provided definitive confirmation of what they had often intuitively sensed, which placed them in a vulnerable situation.

#### 4.2.1 Sub‐Theme 4.2.1: Experiences of Trust in Healthcare Professionals

Experiences of trust in healthcare professionals were described as being treated with respect and understanding, and as being recognised as a person with unique needs and integrity. Clear communication without clichéd phrases and sensitive, respectful, and empathetic behaviours were mentioned as facilitators.

*She was so good, the nurse, I felt she understood. She didn't use clichés or try to touch me. She said she would do anything for us so we could see our daughter*. (3)

Further, Reflections upon consent for autopsy, tissue, and/or organ donation revealed that these were experienced as difficult to reason or decide on in connection with the death notification. However, if they knew the wishes of the deceased, this was described as having facilitated their decision‐making.

How the deceased family member was cared for after their relative's death, how the room was arranged, and the information they were given about the appearance of the deceased person before entering the room were described as affecting participants' experiences. For some deceased individuals, a post‐mortem forensic examination was necessary, which reduced the relatives' ability to touch their deceased family member. This could be perceived as unmanageable. If it were possible to touch a hand, for example, this could ease their experience. When saying farewell to a beloved person, time was described as crucial. Time was needed to grieve, touch, look at, and talk to the dead person. The ability to grieve, alone or together with other relatives, with support from healthcare professionals, facilitated relatives' experiences of their farewell to their family member. Conversely, unclear communication, lack of respectful behaviour, and/or lack of compassion were expressed as barriers to trust in healthcare professionals. After leaving the emergency ward, the ability to receive emotional, informational and instrumental support from healthcare was experienced as reduced or non‐existent, and participants felt a sense of loneliness, not knowing how to handle their emotional reactions or practical difficulties during the grieving process.

#### 4.2.2 Sub‐Theme 4.2.2: Difficult Stances in a Vulnerable Situation

Being asked about family members' attitudes towards organ/tissue donation in connection with death notification was described hypothetically as both overwhelming and difficult for relatives to confront. Some said that they would have needed more time to reason on the deceased's known or presumed wishes or did not want to be asked for this in the immediate aftermath of death. Conversely, others assumed that questions about their family member's attitude towards organ donation should be asked immediately after death. Some said that if they had been informed about others' need for organs, it could have made their reasoning easier.

*In this situation, it would have been difficult to decide about it, if someone had told me about other people's need for organs, maybe it would have been easier?* (10)

Relatives who had previously discussed attitudes towards organ and/or tissue donation within the family assumed that their awareness of the deceased person's wishes could have facilitated their reasoning for their family members' wishes for organ donation if the opportunity to donate had existed. The relatives reflected that this could have made it easier to cope with organ‐preserving medical interventions, because they felt it was important to follow their family member's wishes However, to ‘see with one's own eyes’ that the person was dead, being given enough time to say goodbye, and being promised that medical interventions would not harm or be uncomfortable for their dead family member were wishes mentioned by relatives. Relatives frequently mentioned that an attitude of ‘something good would come out of the worst’ facilitated their reasoning. In contrast, one woman mentioned that her family member's wish had been to donate his/her body to medical research, but for her it felt unmanageable to cope with. She decided to refuse this for her own sake and that of her family. Another woman described how a nurse had called her one hour after her husband's death to ask for her consent to donate tissue from his body. She was surprised and became very upset about this sudden call. She also questioned why she should be asked for consent because the deceased had registered as positive towards organ and tissue donation in the Swedish National Donor Register, and she felt no right to question it. Furthermore, she found the predetermined questions about her husband's lifestyle habits strange and some of them impossible to answer.

*The physician called me, it was a short call, he just informed me about my husband's death and asked if I was alone, nothing more. It was the COVID pandemic, so I couldn't go to the hospital. One hour later, a nurse called me and asked me to decide about tissue donation. She asked strange questions; I was very shocked and upset*. (5)

For some, deciding on an autopsy could be perceived as unmanageable immediately after the death was announced; some wanted this question to be asked later and/or introduced with compassion.

*We sat in the waiting room as if we had broken a hand or something. The physician came and asked if I wanted them to do an autopsy on my husband, I didn't know but I answered yes*. (7)

*When they talked about the autopsy, I reacted something like this, ‘no they're not allowed to cut into my daughter's body.’* (9)

However, in retrospect, the relatives expressed that receiving an explanation of the cause of death was important, and an autopsy was therefore considered important to them.

### 4.3 Theme 3: Balancing a Major Life Change

The loss of a loved one and the grief that followed were experienced as a major life change, which was challenging to process.

#### 4.3.1 With Sub‐Theme 4.3.1: Sense‐Making

The participants described how they reviewed the sequence of events from before the cardiac arrest until the present moment (why, how, and when), trying to find explanations for the death. Both self‐directed guilt and guilt attributed to others were recurrently ruminated upon as participants attempted to determine whether anything could have been done differently to alter the outcome. Lifestyle habits, as well as whether any early signs of illness had been present, were also reviewed.

*He was a skier and in very good shape and we lived healthily. I wish I hadn't gone to bed before him that night, but maybe it wouldn't have changed anything?* (13)

Having had the experience of everything possible being done to save their family member's life was expressed as having an impact on these experiences.

*They'd broken her ribs. I cried because I understood that they'd done everything possible to save her life*. (3)

The autopsy report was perceived as a source of sense‐making and clarification of whether there had been any hereditary factors that could have predisposed the deceased to sudden death. An unidentified cause of death, even following a completed autopsy, was described as a significant barrier to sense‐making. In cases where the cause remained unknown, relatives devoted substantial time to trying to understand why the death had occurred.

#### 4.3.2 Sub‐Theme 4.3.2: Sharing Grief

Relatives described how grief was shared with other relatives, close friends and/or people with similar experiences and/or those who listened compassionately. Sometimes, expressions of grief differed between relatives, which could be experienced as an obstacle within the family. In contrast, some relatives reported that they were afraid of causing pain to other, more sensitive relatives and therefore withheld information about their own experiences. Both different expressions of grief within the family and withholding were experienced as leading to loneliness.

*We talked a lot about him but not about how he died. I think that's because it's hard for me to talk about that and I think they find it hard to know*. (1)

Sometimes, former friends withdrew, and others avoided responding or confronting the grieving person. One woman said:

*People get scared of me. When they meet me, you can see the fear in their eyes, I became like a pariah to others*. (1)

Informing others about the death was often carefully planned to reduce harm and was expressed as a responsibility for the well‐being of others. Information might be shared via Facebook, phone calls or visits to other relatives. Sometimes, the appropriate moment for delivering the death notification was carefully chosen, even if this meant that the notification was deliberately delayed.

#### 4.3.3 Sub‐Theme 4.3.3: Need for Support

Although family, friends and/or superficial acquaintances could be perceived as supportive, family members often described a lack of support from healthcare providers. It could be experienced as difficult to determine where to ask questions and how to get practical, informative, mental and/or psychological support

*As we left the hospital, I thought about what's the next step, what happens now, what are we going to do? The counsellor at the hospital could have informed us about that*. (11)

For mental and/or psychological support, some participants described finding their own solutions by contacting bereavement groups, deacons, their employer, a psychologist or a counsellor. However, when addressing practical issues, relatives often struggled on their own.

#### 4.3.4 Sub‐Theme 4.3.4: Self‐Comfort and Dispersing Negative Thoughts

The participants reported strategies that were used for self‐comfort and to distract themselves. These included: reprioritising one's life and lifestyle, for example, spending more time with the family; reducing working hours; continuing to work; or engaging in activities that provided relaxation, such as physical activities and travelling. For some relatives, it was consoling that the death had occurred suddenly, as this was understood to indicate that the deceased had not experienced suffering. Other strategies for self‐comfort were described as the assumption that the person died at the peak of their life and career, was happy when the death occurred, and that their life together had been happy despite great sadness and loss. One woman said:

*We had a very good life together, so I've escaped that kind of brooding. Although I am very sad that we didn't get a longer life together*. (2)

Several relatives mentioned that a worse outcome would have been brain damage caused by oxygen deficiency during the cardiac arrest. One son shared his thoughts:

*Seven minutes without a pulse, what did that do to the brain and the body? It probably would have been worse for him and for us, but strangely enough this could be processed*. (10)

## 5 Discussion

This study aimed to explore relatives' experiences of a family member's circulatory death after out‐of‐hospital cardiac arrest and to explore their hypothetical perspectives on being asked to decide about organ donation in this situation. The analysis generated three themes: ‘Fluctuating between hope and despair’, which describes relatives' experiences of their family member's sudden cardiac arrest; ‘Exposed to vulnerability’, which concerns their experiences of death and perspectives on reasoning about the deceased persons wishes regarding donation; and ‘Balancing a major life change’, which involves experiences of facilitators and barriers to managing the loss.

Implementing an uncontrolled donation after circulatory death process poses significant ethical challenges, particularly when the preferences of the deceased are unclear to their relatives; however, determining the optimal course of action in the patient's best interest remains essential (Georgiou et al. 2025). Participants in this study described healthcare professionals´ competence in compassionate behaviours and communication skills as factors that could influence their ability to interpret the wishes of the deceased, as well as to cope with their overall experiences of their family member's death. As described in previous research, assumptions about trust between patients/families and healthcare professionals are based on considerations of integrity, reliability, and having benevolent intentions towards the patient/relative (Armstrong 2024), which is consistent with the findings of this study. Additionally, in the implementation of an uncontrolled donation after circulatory death process, timing is crucial for the preservation of organs (Georgiou et al. 2025). Nevertheless, relatives in this study expressed a strong need to be given sufficient time to say goodbye to their family member. This raises the question of whether such time could be provided after the donation has been completed. Although the participants were aware that their family member was dead, they reflected on the importance of being assured that the donation procedure would not cause any pain or discomfort to their family member. Additionally, despite their awareness of death, it was important for relatives that healthcare professionals respected and cared for the deceased as if the person were still alive. A previous systematic review of family members' experiences following organ donation consent for donations after brain death or donations after circulatory death described the complexity of decision‐making in line with the deceased person's wishes, whereas ambivalence, uncomfortable conversations and supportive needs afterwards were attributes that affected family members' experiences (Ma et al. 2021). Furthermore, Sque et al. described how prior knowledge, experiences, attitudes, beliefs and one's own intentions towards organ and tissue donation influenced family members' decision‐making regarding donations after brain death and donations after circulatory death (Sque et al. 2018). In a qualitative study by de Groot et al., bereaved relatives of eligible brain‐dead donors stated that reasons for refusing consent were attributed to contextual factors such as being overwhelmed by the notification, lack of support, and lengthy procedures (de Groot et al. 2016). This is in line with the findings of our study and suggests common experiences and needs among relatives regardless of how the death occurred or the donation method that might be implemented. Sharing grief with other relatives, friends, and more superficial acquaintances was experienced as a source of social support through the grieving process, but for many participants in this study, professional support was needed. In fact, the need for support could be linked to social networks and coping skills, which have also been stated in previous research (Carlsson, Alvariza, et al. 2022), and professional support while learning to deal with grief may be necessary (Thomson et al. 2024). A recent study of the psychological needs of relatives affected by young sudden cardiac death and the impact of social support found that this kind of support offered a safe environment, supported sense‐making and facilitated a new sense of meaning for families (Steffen et al. 2020). However, both professional support and social support must be proactive and extend over time (Carlsson, Alvariza, et al. 2022). Participants in this study frequently mentioned a lack of support from healthcare providers after they had left the emergency department. Experiencing a lack of support from healthcare providers was also a finding in a previous meta‐synthesis of family members' needs following death from sudden cardiac arrest (Zhang et al. 2024), This is largely similar to findings in previous research into relatives' experiences after sudden cardiac arrest (Carlsson, Bremer, et al. 2022; Douma et al. 2023; Zhang et al. 2024). These findings suggest that follow‐up is essential for relatives following both death in out‐of‐hospital cardiac arrest and organ donation. However, uncontrolled donation after circulatory death presents additional challenges related to the time constraint required for organ preservation and the communication with relatives (Georgiou et al. 2025). These aspects must be carefully considered to optimise outcomes regarding relatives' well‐being, the deceased's wishes, and retrieval of the organs.

### 5.1 Strengths and Limitations of the Work

A limitation in this study could be attributed to the small sample size (13 participants). However, data collection and coding continued until no new data, additional insights, or patterns emerged during the analysis, which was a strength of this study. Furthermore, Patient and Public Involvement was not incorporated in the design, analysis, or reporting of the study. Such involvement could have contributed to a different structure for the study and provided unique perspectives and additional validation of the themes. Moreover, the recruitment of participants via advertisements might have influenced the sample because people who are positive about donation may have been more likely to participate. However, three participants were recruited by direct request and therefore did not actively choose to participate. Regardless of recruitment method, the participants' experiences and reflections varied. Additionally, the time interval between the family member's death and the interview may present a risk of recall bias. However, the relatives provided detailed and rich narratives of their experiences regardless of the time that had passed. Another limitation was that the participants in this study lived in a small country with high socioeconomic standards and good public healthcare. Findings from other contexts might have identified different themes, as the analysis was influenced by contextual and situational factors. Therefore, the variety of experiences and reflections reported in this study may have been less common in other cultures and contexts, which could influence the transferability of the findings.

### 5.2 Recommendations for Further Research

To provide comprehensive, high‐quality nursing and care for relatives, further research is needed on family members' experiences following uncontrolled donation after circulatory death, with particular attention to contextual factors, communication, and relatives' specific needs. In addition, future research should explore ethical dilemmas and propose strategies developed through dialogue with the public and with health‐care professionals involved in the care of individuals who die from out‐of‐hospital cardiac arrest and who may be considered potential candidates for uncontrolled donation after circulatory death.

### 5.3 Implications for Policy and Practice

Policies and clinical practice should ensure that relatives are adequately informed and supported in reasoning on their family members' wishes regarding organ donation. To achieve this, nurses and other healthcare professionals require training in compassionate and empathetic communication strategies. Such training is essential both for guiding relatives through reasoning about their family members' preferences and for providing emotional support during the grieving process.

## 6 Conclusions

Awareness of the deceased family member's wishes regarding organ donation could facilitate relatives' ability to support the deceased's known or presumed wishes. When such wishes of the deceased are unknown to the relatives, determining the optimal course of action in the patient's best interest must be carefully considered. The ability of healthcare professionals to demonstrate compassion and empathy, and communicate clearly and respectfully, appears to influence relatives' capacity to reason about the deceased's wishes as well as their ability to cope with their loss. A follow‐up that combines both informational and emotional support may benefit relatives' emotional processing of their loss. Such support should adhere to established protocols and involve trained personnel to ensure both professional and compassionate care, consistent with family‐centred principles. Additionally, relatives often express the need for sufficient time to say goodbye. While organ preservation requires precise timing, these needs must be thoughtfully considered to determine how they can be optimally accommodated in practice.

## Funding

This study is part of a larger project investigating the conditions for uDCD in Stockholm/Sweden from multiple perspectives. The entire project is funded by Region Stockholm, which is responsible for healthcare in Stockholm County.

## Conflicts of Interest

The authors declare no conflicts of interest.

## Supporting information


**Supplementary Table 1:** Descriptions of themes with illustrative quotes.

## Data Availability

The data that support the findings of this study are available from the corresponding author upon reasonable request.
