# Supplementary material for: Relatives' Experiences of Circulatory Death Following Out‐of‐Hospital Cardiac Arrest and Their Hypothetical Perspectives on Organ Donation: A Qualitative Study
Source: J Adv Nurs. 2026 Apr 5;82(11):9883–92. doi: 10.1111/jan.70603 (PMC13661776; doi:10.1111/jan.70603)
Supplement: Supplementary file 1 [file JAN-82-9883-s001.docx]

**Supplementary Table 1. Descriptions of themes with illustrative quotes.**

| **Theme** | **Sub-themes** | **Description of themes** | **Illustrative quotes** |
| --- | --- | --- | --- |
| **Fluctuating between hope and despair** |  | During this theme, relatives described their extremely stressful experiences and feelings of hope and despair related to their loved one’s sudden cardiac arrest. In addition, feelings of panic and loneliness were described. Sensitive and empathic behaviours from the rescue personnel or other people were perceived as support. Experiences where everything possible had been done to save their loved one’s life facilitated processing afterwards. | *‘He had run and sat on the stairs. I was glad he was home. It became so quiet so I looked at him and he was lying down by the stairs, I thought he was joking but then I shook him and he was completely gone.’ (2)*  *‘As I was standing there during the CPR I thought “I hope he wakes up” or “I hope not” because he’s probably very brain damaged. You’re like that all the time.’ (1)*  ‘*The rescue manager stayed for a very long time and talked to me, it felt very good.’ (5)* |
| **Exposed to vulnerability** | **Experiences of trust in healthcare professionals**  **Difficult stances in a vulnerable situation** | This theme describes relatives’ experiences and emotional reactions and needs when hope is gone and their loved ones are declared dead. Factors such as healthcare professionals’ ability to communicate and their behaviours affected relatives. Being treated as an object/subject influenced relatives’ experiences and having enough time to say farewell is crucial for processing afterwards. In addition, relatives often experienced an abrupt interruption of emotional, instrumental and informational support when they left the hospital, which evoked a feeling of loneliness.  In this theme, relatives also described their experiences, needs and requirements to take difficult decisions about organ and/or tissue donation, donation to medical research and/or autopsy.  Deciding whether to authorise an autopsy or organ or tissue donation feels difficult and sometimes overwhelming in connection with the death notice. However, knowing the wishes of the deceased facilitated this and made relatives more prepared to take a stand on tissue and/or organ donation. Nevertheless, relatives hypothesised that such a decision could imply something good emerging out of misery. | *‘She was at the hospital so I thought it would go well, but when I entered the room at the hospital, I understood that she was gone.’ (7)*  *She was so good, the nurse, I felt she understood. She didn’t use clichés or try to touch me. She said she would do anything for us so we could see her.’ (3)*  *‘The physician called me, it was a short call, he just informed me about my husband’s death and asked if I was alone, nothing more. It was the COVID pandemic so I couldn’t come to the hospital. One hour later a nurse called me and asked me to decide on tissue donation. She asked strange questions, I was very shocked and upset.’ (5)*  *‘We sat in the waiting room as if we had broken a hand or something. The physician came and asked if I wanted them to do an autopsy on my husband, I didn’t know but I said yes.’ (7)*  *When they talked about the autopsy, I reacted something like this: “no they can’t cut into my daughter’s body”.’ (9)*  *‘In this situation it would be hard to decide, if someone had told me of others’ need for an organ, might it have been easier?’ (10)* |
| **Balancing a major life change** | **Sense-making**  **Sharing grief**  **Need for support**  **Using self-comfort and dispersing negative thoughts** | This theme outlined how relatives struggled to manage and understand the meaning of their loss and a different everyday life. Furthermore, a feeling of loneliness despite support from the environment must be managed. This processing means struggling with emotional reactions, practical burdens, responsibility for others, a sense of guilt, others’ guilt and their need for emotional, informational and instrumental support. Experiencing a lack of support from healthcare professionals could be hard to process and left a feeling of loneliness. To facilitate processing, different strategies for self-consolation and dispersing thoughts could be used. | *They ‘d broken her ribs. I cried because I understood that they’d done everything possible to save her life.’ (3)*  ‘*As we left the hospital, I thought about: what’s the next step, what happens now, what are we going to do? The counsellor at the hospital could have informed us about that.’ (11)*  *‘He was a skier and in very good shape and we lived healthily. I wish I hadn’t gone to bed before him that night, but maybe it wouldn’t have changed anything?’ (13)*  *We talk a lot about him but not about how he died. I think that’s because it’s hard for me to talk about that and I think they find it hard to know.’ (1)*  *‘Seven minutes without a pulse, what did that do to the brain and the body? It probably would have been worse for him and for us, but strangely enough this could be processed.’ (10)*  *‘We had a very good life together, so I’ve escaped that kind of brooding. Although I am very sad that we didn’t get a longer life together.’ (2)*  *‘People get scared. When they meet me you can see the fear in their eyes. I became like a pariah to others.’ (1)* |
